# Unprecedented Ultraviolet Circularly Polarized Light‐Dependent Anomalous Photovoltaics in Chiral Hybrid Perovskites

**DOI:** 10.1002/advs.202412506

**Published:** 2025-01-15

**Authors:** Qianwen Guan, Peng Xu, Bohui Xu, Huang Ye, Zeng‐Kui Zhu, Shiyu Wang, Chengshu Zhang, Hang Li, Chengmin Ji, Zheshuai Lin, Junhua Luo

**Affiliations:** ^1^ State Key Laboratory of Structure Chemistry Fujian Institute of Research on the Structure of Matter Chinese Academy of Sciences Fuzhou Fujian 350002 P. R. China; ^2^ University of Chinese Academy of Sciences Beijing 100049 P. R. China; ^3^ Functional Crystals Lab Technical Institute of Physics and Chemistry Chinese Academy of Sciences Beijing 100190 P. R. China

**Keywords:** anomalous photovoltaic effect, chiral hybrid perovskites, circularly polarized light detection

## Abstract

Circularly Polarized Light (CPL)‐dependent anomalous photovoltaic effect (APVE), characterized by light helicity‐manipulated steady photocurrent and above‐bandgap photovoltage, has demonstrated significant potential in the fields of photoelectronic and photovoltaics. However, exploiting CPL‐dependent APVE in chiral hybrid perovskites, a promising family with intrinsic chiroptical activity and non‐centrosymmetric structure, remains challenging. Here, leveraging the flexible structural design of chiral alternating cations intercalation‐type perovskites, CPL‐dependent APV, for the first time, is achieved in chiral perovskites. Specifically, by introducing lone pair electrons into the organic layers to greatly amplify the polarization, [(R)‐PPA](MOPA)PbBr_4_ (**2‐R**) (PPA = 1‐phenylpropylammonium, MOPA = 3‐methoxypropylammonium) exhibit intrinsic APVE with an above‐bandgap photovoltage of 6.50 V (*E*_g_ = 3.01 eV) under ultraviolet (UV) light illumination. Strikingly, profiting from the natural chiral optical activity of chiral perovskites, unprecedented UV CPL‐dependent APV is realized in **2‐R**, driving the high distinguishability between right‐hand and left‐hand CPLs with a large anisotropy factor (*g*_Iph_) of 0.33. This study pioneers the realization of CPL‐dependent APV within chiral perovskite, promising significant advancements in optoelectronic device technologies.

## Graphical abstract

By introducing achiral large dipole moment cations into the organic layers to greatly amplify the polarization, 2D [(R)‐PPA[MOPA]PbBr_4_ (**2‐R**, PPA = 1‐phenylpropylammonium, MOPA = 3‐methoxypropylammonium) shows intrinsic anomalous photovoltaics in chiral perovskites. Strikingly, based on the natural chiral optical activity and anomalous photovoltaics, **2‐R** shows unprecedented ultraviolet CPL‐dependent anomalous photovoltaics, shedding light on future practical applications.

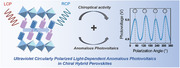

## 1 Introduction

The bulk anomalous photovoltaic effect (APVE) in non‐centrosymmetric materials can generate a photovoltage greater than their bandgap, which can promote the separation of photogenerated electronic‐hole pairs and improve the photoelectric conversion efficiency for the photovoltaic device.^[^ ^1^ ^]^ In this context, profiting from materials’ nonlinear response to light absorption and electron transitions, the APVE can be manipulated by the state of polarization of light, including linearly polarized (LPL)^[^ ^2^ ^]^ and circularly polarized light (CPL).^[^ ^3^ ^]^ Among them, CPL‐dependent APVE, wherein the open‐circuit voltage and short‐circuit current exhibit sensitivity to the light's helicity, has garnered significant attention in spintronics, quantum information processing, and optoelectronics.^[^ ^4^ ^]^ Generally, the absence of inversion symmetry is the essential factor for the CPL‐dependent APVE, which influences the spin behavior of charge carriers and results in differential absorption for left‐ and right‐handed CPL and asymmetric photovoltaic responses.^[^ ^5^ ^]^ Initially, the CPL‐dependent APVE was exploited in traditional inorganic material. For example, based on helicity‐dependent interaction between light and domain variants,^[^ ^6^ ^]^ noncentrosymmetric BiFeO_3_ shows intrinsic CPL‐dependent APVE, which allows a switch‐like APVE by changing the helicity of CPL.^[^ ^3b^ ^]^ However, traditional inorganic photoelectric detectors generally require complicated fabrication techniques and high costs, showing the necessity of exploiting novel, economically friendly material systems.^[^ ^7^ ^]^

Organic–inorganic hybrid perovskites (OIHPs), characterized by their simple fabrication process, inherent structural tunability, and excellent semiconductor properties, have emerged as promising candidates for CPL‐dependent APVE materials.^[^ ^8^ ^]^ For instance, Zhu et al. were the first to achieve CPL‐dependent APVE in OIHPs by utilizing the chiroptical activity of non‐centrosymmetric perovskites, (4‐AMP)BiI₅ (4‐AMP = 4‐(aminomethyl)piperidinium). This material exhibited remarkable CPL differentiation capabilities, as evidenced by a significant asymmetry factor of 0.24.^[^ ^3a^ ^]^ However, this rare CPL‐dependent APVE material crystallizes in the achiral m space group, necessitating alignment along the orientation of the screw optical axis to elicit chiroptical signals, which enhances the difficulty of CPL detection. Therefore, it is urgent to develop direct CPL‐dependent APVE materials within OIHPs.^[^ ^9^ ^]^ Fortunately, recent advancements have shown that inserting chiral cations into the inorganic framework enables the construction of intrinsic non‐centrosymmetric chiral OIHPs.^[^ ^10^ ^]^ More importantly, the chiral OIHPs show natural chiroptical activity without screw optical orientation restriction, which is expected to be a promising direct CPL‐dependent APVE material to exploit sensitive self‐driving CPL detection performance.^[^ ^8^, ^10^, ^11^ ^]^ However, until now, the realization of CPL‐dependent APVE effects in chiral perovskites is still challenging.

Here, leveraging the burgeoning structural design of chiral alternating cation intercalation (ACI) perovskites, CPL‐dependent APVE was first realized in chiral perovskites. Specifically, by introducing the achiral cation with lone pair electrons into the organic layers, the polarization was significantly enhanced in (R‐PPA)(MOPA)PbBr_4_ (**2‐R**) (PPA = 1‐phenylpropylammonium, MOPA = 3‐methoxypropylammonium), compared with (R‐PPA)(PA)PbBr_4_ PA = propylammonium). Surprisingly, under 377 nm light, **2‐R** exhibits APVE with a high open‐circuit voltage (*V*_oc_) up to 6.50 V, exceeding the optical bandgap (*E*_g_ = 3.01 V), unprecedented in chiral perovskites. Profiting from the APVE, **2‐R** exhibits excellent photo‐response and stable photocurrent with negligible change under long exposure and multiple on/off cycles. More importantly, based on the intrinsic chiroptical activity, **2‐R** exhibits unprecedented UV CPL‐dependent APV, facilitating self‐driven CPL detection with high photocurrent anisotropy factors (*g*_Iph_) of 0.33 at zero bias. This work fills the CPL‐dependent APVE's blank in chiral‐polar perovskites and lays the material foundation for developing high‐performance optoelectronic devices.

## 2 Results and Discussion

### 2.1 Basic Crystal Characterization

**Figure 1:**
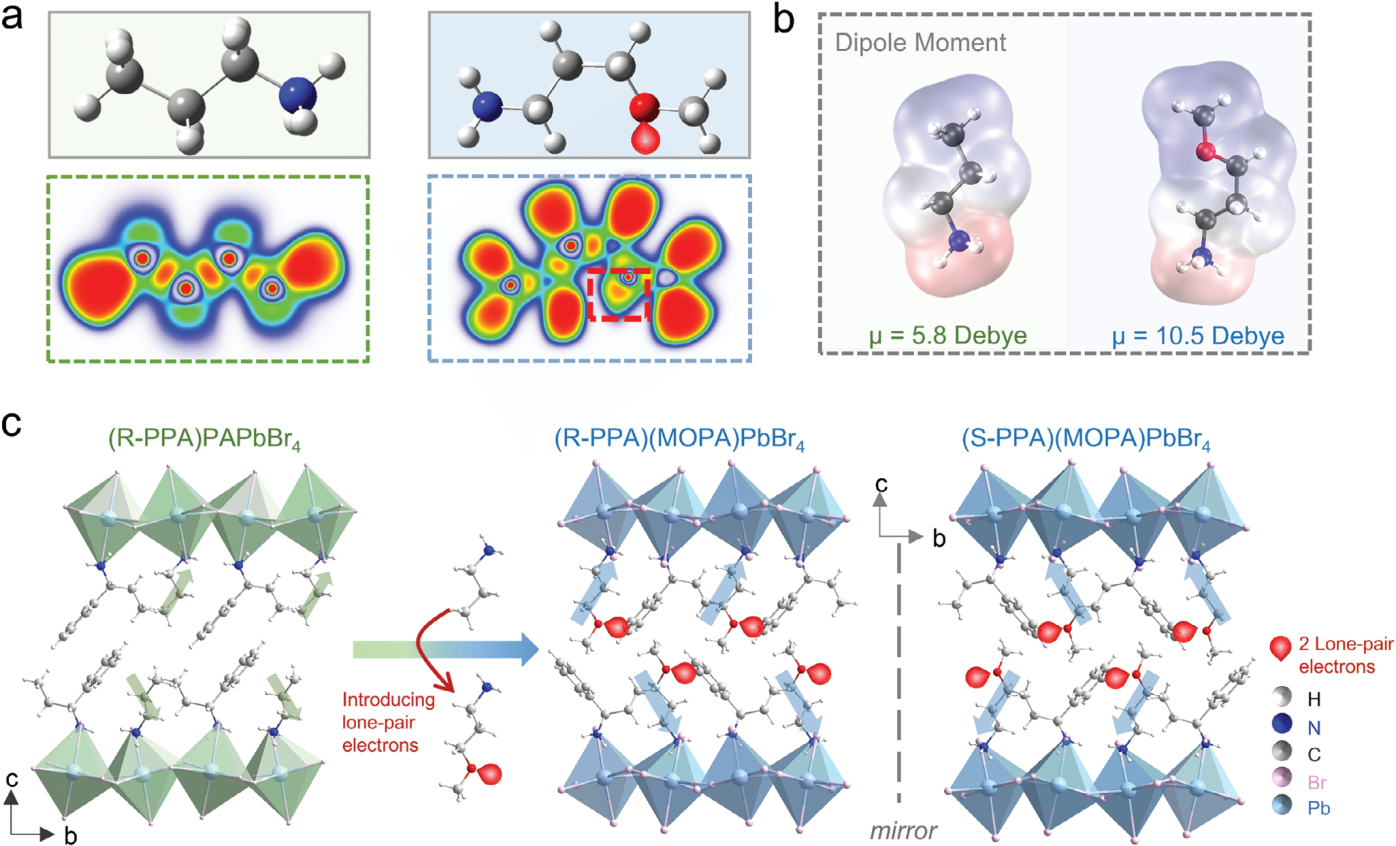
The design strategy of ACI chiral perovskite structure. a) The ELF spectra of PA and MOPA molecules present the unique lone‐pair electrons of MOPA. b) the dipole moment calculation of PA^+^ and MOPA^+^ cations. c) MOPA cations substitute PA in (R‐PPA)PAPbBr_4_ **1‐R** to design (R‐PPA)(MOPA)PbBr_4_ **2‐R** and its enantiomer (S‐PPA)(MOPA)PbBr_4_ **2‐S** (The 2 lone‐pair electrons refer to the two pairs of lone‐pair electrons on O atoms).

Generally, introducing a group with highly electronegative lone‐pair electrons into an organic amine can disrupt the charge distribution, resulting in increased polarity across the molecule.^[^ ^12^ ^]^ **Figure** **1**a shows the chemical structures and electronic local function (ELF) maps of PA (propylamine) and MOPA (3‐methoxypropylamine). The ELF maps present MOPA with significant lone‐pair electrons on the O atom in a half‐moon shape.^[^ ^13^ ^]^ To visualize the asymmetric distribution of charge by introducing a CH_3_O‐ group, the electrostatic potential (ESP) maps of MOPA and PA are shown, where MOPA presents a larger molecular dipole moment (μ = 2 Debye) than that of PA (μ = 1.4 Debye) (Figure S1, Supporting Information). Significantly, these organic amine molecules are usually present in a protonated state in the crystal structure, which leads to a larger dipole moment due to the protonation inducing increased asymmetry in the internal charge distribution of the molecule (Figure 1b. μ(PA^+^) = 5.8 Debye, μ(MOPA^+^) = 10.5 Debye).^[^ ^14^ ^]^ In this context, utilizing the structural flexibility of chiral alternating cation intercalation (ACI) perovskites, we replace PA with MOPA, which, for the first time, introduces a cation with lone‐pair electrons into chiral ACI perovskites (Figure 1c). More significantly, incorporating cations with large dipole moments significantly influences crystal polarity and modifies nonlinear optical properties. Specifically, a pair of novel chiral ACI perovskites (R‐/S‐PPA)(MOPA)PbBr_4_ (**2‐R/S**, PPA = 1‐phenylpropylamium) were successfully synthesized by reacting the stoichiometric amounts of MOPA, R‐/S‐PPA, and Pb(Ac)_2_·3H_2_O in hydriodic acid HBr (details in the Supporting Information). Utilizing single‐crystal X‐ray diffraction, the structure of **2‐R** and **2‐S** are obtained, which crystalized in a chiral polar space group (*P*2_1_) with intrinsic chiroptical activity and nonsymmetric structure, laying the foundation for generating CPL‐dependent anomalous photovoltaic effect (APVE) (Table S1, Supporting Information).^[^ ^3a^ ^]^ Their phase purity was demonstrated by powder X‐ray diffraction (PXRD), where the experimental spectra matched well with the simulated one (Figure S2, Supporting Information). ^[^ ^15^ ^]^ Based on the crystal structure of **2‐R**, the dipole moment of the inorganic framework, organic cations, and total crystal structure are calculated (Figure S3, Supporting Information). As shown in Table S2 (Supporting Information), due to the introduction of large dipole moment cations (MOPA), **2‐R** shows a larger dipole moment (23.5833 Debye) than **1‐R** (7.5568 Debye). Strikingly, the large dipole moment of the crystal structure can induce significant spontaneous polarization, which shows potential for outstanding polar performance.^[^ ^16^ ^]^

**Figure 2:**
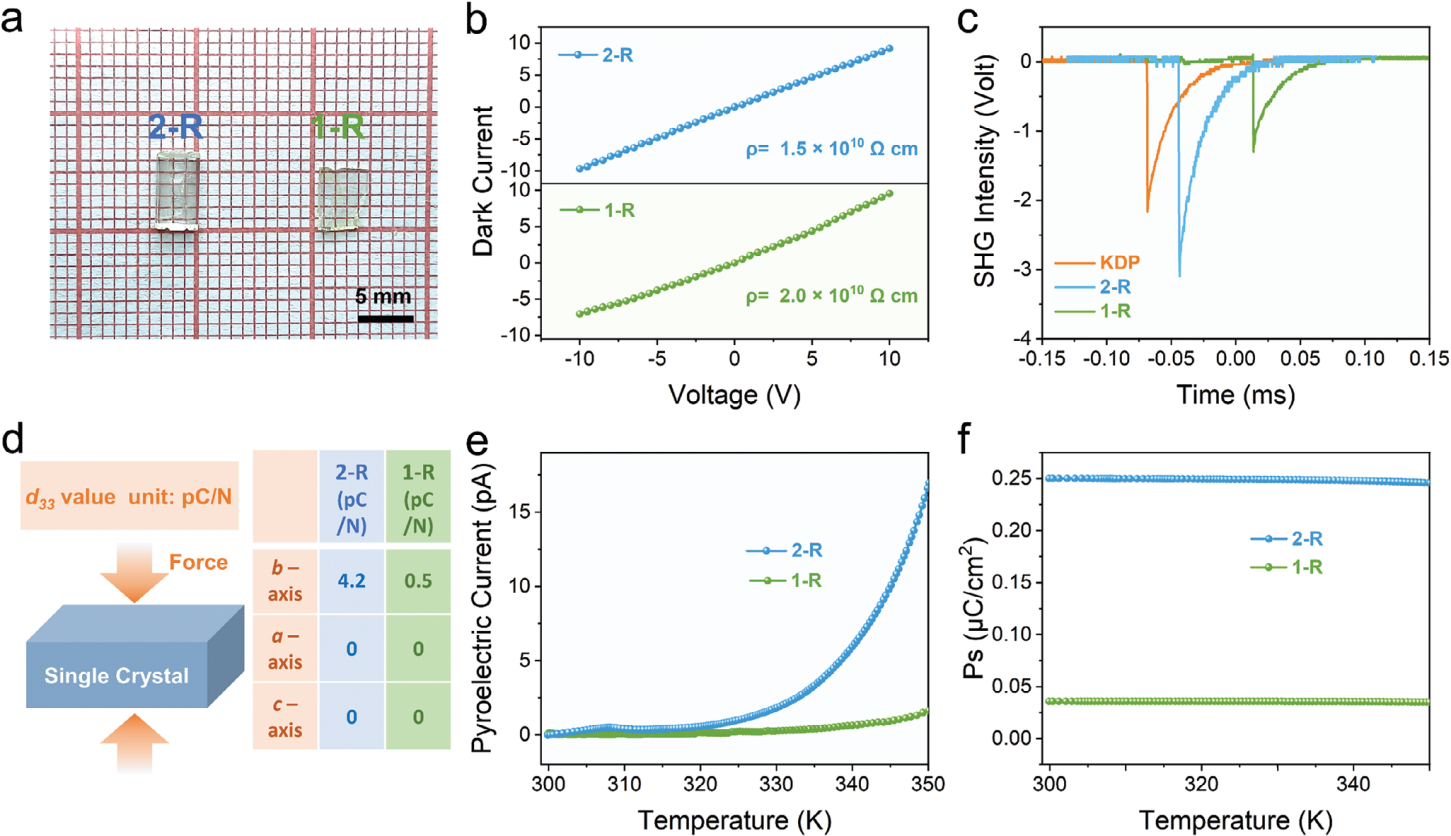
Crystal polarity characterization. a) The bulk single crystal of **2‐R** and **1‐R**. b). The resistivity of **1‐R** and **2‐R** along the *b*‐axis. c). The second harmonic generation of **1‐R** and **2‐R**. d). The piezoelectric performance of **2‐R** and **1‐R**. e). The pyroelectric performance of **2‐R** and **1‐R**. f). The spontaneous polarization values of **2‐R** and **1‐R**.

Based on such an experimental conception, the bulk single crystals (SC) of **1‐R** and **2‐R** with a size of 6 × 3 × 1 mm^3^ and 5 × 4 × 0.5 mm^3^ have been respectively obtained by slowly cooling programmed cooling (**Figure** **2**a, Details are shown in the Supporting Information). The quality of the SC was characterized using scanning electron microscopy (SEM), atomic force microscopy (AFM), and resistivity measurements. As shown in Figure S4 (Supporting Information), SEM and AFM spectra present the flat and smooth planes of **1‐R** and **2‐R** SC, reflecting their few surface defects, conducive to carrier mobility and the device's photoelectric conversion efficiency.^[^ ^17^ ^]^ Moreover, the SC devices of **1‐R** and **2‐R** show large bulk resistivity (ρ) of 1.5 × 10^10^ and 2.0 × 10^10^ Ω cm, respectively, ≈10^2^ times higher than those of 3D lead halide perovskites (10^7^–10^8^ Ω cm)^[^ ^18^ ^]^ (Figure 2b). Such a large bulk resistivity reflects their high crystal quality, which is advantageous for minimizing dark and noise currents to achieve high‐performance photoelectric detection.^[^ ^19^ ^]^

### 2.2 Polar Structure‐Induced Related Properties

Based on the high‐quality SC, the non‐centrosymmetric and polarization performance of **1‐R** and **2‐R** are explored by second harmonic generation (SHG), piezoelectric, and variable‐temperature pyroelectric measurement. As expected, **2‐R**, with its larger dipole moment, shows stronger SHG signals (1.3 KDP) than **1‐R** (0.5 KDP), indicating a more asymmetric structure of **2‐R** (Figure 2c; measurement details are shown in the Supporting Information).^[^ ^20^ ^]^ Then, their piezoelectric performance was explored based on **1‐R** and **2‐R** SC. As shown in Figure 2d,**2‐R** exhibits a larger piezoelectric response along the polar axis, with a direct piezoelectric coefficient (*d*_33_) value of 4.2 pC/N, compared to **1‐R** (0.5 pC/N). This demonstrates the more non‐centrosymmetric crystal structure of **2‐R**, which enables it to generate more charges in response to mechanical forces.^[^ ^21^ ^]^ Meanwhile, **2‐R** displays remarkable variable‐temperature pyroelectric behavior along the polar axis, in which the current enhances with the increased temperature under zero bias (Figure 2e). Based on the current‐temperature curve, the polarization value (*P*_s_) is estimated (Calculation details are shown in the Supporting Information).^[^ ^22^ ^]^ As shown in Figure 2f, **2‐R** has a larger *P*_s_ value of 0.25 µC cm^−2^ than **1‐R** (0.03 µC cm^−2^) at room temperature. Consequently, owing to introducing a large dipole moment cation (MOPA), **2‐R** shows significant polarization, which shows its tremendous potential for generating a large built‐in electric field for self‐driven photoelectric detection.

### 2.3 CPL‐Dependent Anomalous Photovoltaics

**Figure 3:**
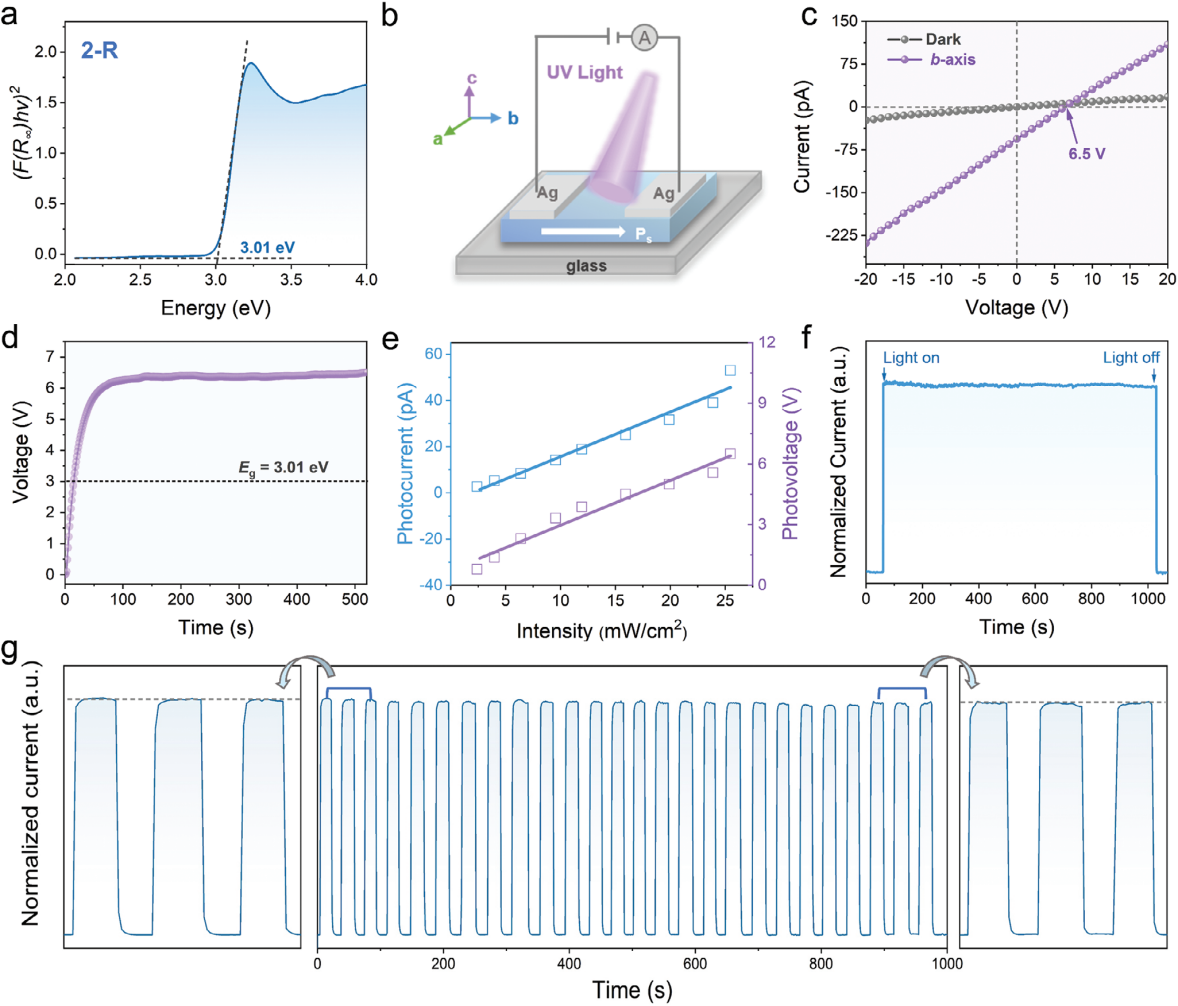
Photoelectric measurements of **2‐R** under 377 nm laser. a) The optical bandgap of **2‐R** is calculated by the *Tauc* equation. b) The diagram of photoelectric measurements. c) The *I*‐*V* curves of **2‐R** in the dark and light along the *b*‐axis. d) The steady‐state *V*_oc_ exceeds the bandgap. e) The *I*_sc_ and *V*_oc_ of **1R** as functions of the light intensity. f) The light stability of **2‐R** g) The switching stability of **2‐R** at zero bias.

Inspired by the excellent polarity properties of **2‐R**, its bulk photovoltaic effect under illumination is expected to be exploited.^[^ ^23^ ^]^ First, according to the UV–vis absorbance spectra, the optical bandgap (*E*_g_ = 3.01 eV) of **2‐R** was calculated by the *Tauc* equation (**Figure** **3a**), which is comparable with the estimated value (2.967 eV) obtained by density functional theory (DFT) method (Figure S5, Supporting Information). The reported absorption edge and bandgap of **1‐R** is 415 nm and 3.01 eV, respectively.^[^ ^24^ ^]^ Based on the bandgap and absorption spectra of **2‐R** and **1‐R**, a 377 nm laser is selected as the incident light to explore its photovoltaic performance along the polar axis (Figure 3b). Surprisingly, under 377 nm with a of 25.5 mW cm^−2^, **2‐R** shows a large photovoltage of 6.50 V (Figure 3c), which exceeds its bandgap (*E*_g_ = 3.01 eV), identifying an apparent anomalous photovoltaic along the polar axis. To our knowledge, anomalous photovoltaics are first realized in chiral hybrid perovskites (Table S3, Supporting Information).^[^ ^8c^ ^]^ The photovoltage of **2‐R** (6.50 V) is significantly higher than **1‐R** (0.4 V) under the same light intensity (Figure S6, Supporting Information). Moreover, as shown in the Voltage–time measurement, the anomalous photovoltage remains stable under illumination over a long time range (Figure 3d), highlighting its reliability for continuous operation in photovoltaic applications. Increasing light intensity, the open‐circuit voltage (*V*_oc_), and short‐circuit photocurrent (*I*_sc_) are linear growth, confirming the linear output of the photodetector (Figure 3e; Figure S7, Supporting Information).^[^ ^25^ ^]^ Unfortunately, limited by the power of our laser device, we cannot obtain the larger and saturated APV value, expecting further exploitation in the future. Nevertheless, the generation of APVE in our material facilitates the dissociation of photo‐excited carriers without an applied bias voltage, which is crucial for enhancing the efficiency of photovoltaic and optoelectronic devices.^[^ ^25^ ^]^ As shown in Figure S8 (Supporting Information), under the same power, the photocurrent of **2‐R** is 5.5 times larger than that of **1‐R**, identifying that the APV can effectively separate the photogenerated carriers. Based on excellent carrier separation and transport capabilities, the **2‐R** exhibits better optoelectronic performance than the **1‐R** (Figure S9, Supporting Information). Meanwhile, benefiting from a stable anomalous photovoltaic drive, the photocurrent shows negligible change under long exposure and multiple on/off cycles (Figure 3f,g), showing its excellent durability in self‐driven photoelectric detection.^[^ ^26^ ^]^

**Figure 4:**
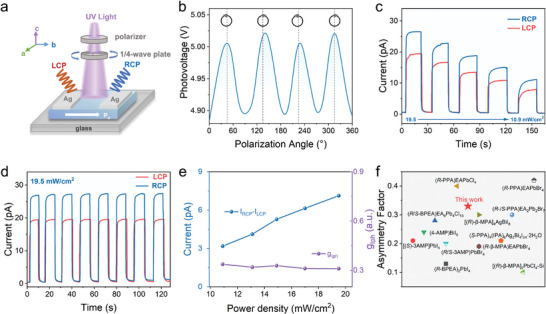
The self‐powered CPL detection of **2‐R**. a) The diagram of CPL detection. b) CPL anormal photovoltage of **2‐R** as a function of the rotation angle. c) The photocurrents of the device under RCP and LCP illumination with different light intensities, respectively. d) The on/off cycle under RCP and LCP illumination e) Current difference between RCP and LCP and asymmetric factor (*g*_Iph_) f) The anisotropy factor of this work in comparison with the reported chiral materials.

Based on the unique APVE and excellent photo‐response, **2‐R** shows tremendous potential for circularly polarized light (CPL) dependent APVE and efficient CPL detection performance.^[^ ^27^ ^]^ The circular dichroism (CD) measurement was carried out to examine the chiroptical properties of **2‐R/S**, which shows the obvious negative and positive CD signals in Figure S10 (Supporting Information).^[^ ^28^ ^]^ Based on its intrinsic chirality and APVE, sensitive self‐driven CPL detection is desirable to exploit for **2‐R**. Passing the 377 nm laser beam through a polarizer and a quarter‐wave plate, CPL can be generated and illuminated onto **2‐R** (**Figure** **4a**).^[^ ^29^ ^]^ Strikingly, **2‐R** exhibits CPL‐dependent APVE under a light intensity of 19.5 mW cm^−2^, in which the *V*_oc_ changes with light's helicity, indicating its excellent circularly polarization‐dependent photoresponse (Figure 4b). The realization of CPL‐dependent APVE is unprecedented in chiral hybrid perovskites, which is critical for the photovoltaic field.^[^ ^3b^ ^]^ Based on the CPL‐dependent APVE, the CPL detection was performed on **2‐R**, which shows excellent differentiation capability and switching stability of RCP and LCP (Figure 4c,d). To evaluate the detectability of circularly polarized light, the anisotropy factor of the photocurrent, *g*_Iph_, is calculated using the equation *g*_Iph_ = 2(*I*_R_^–^ *I*_L_)/(*I*_R_ + *I*_L_), where *I*_R_ and *I*_L_ represent the photocurrents under RCP and LCP illumination, respectively.^[^ ^30^ ^]^ As shown in Figure 4e, **2‐R** presents excellent CPL detection performance under different light intensities with the *g*_Iph_ up to 0.33 under 19.5 mW/cm^2^. As depicted in Table S4 (Supporting Information) and Figure 4f, the anisotropy factor of **2‐R** is larger than most of the reported CPL detectors,^[^ ^31^ ^]^ such as (*R*‐MPA)_4_AgBiI_8_ (0.3),^[^ ^31c^ ^]^ (R)‐β‐ MPA]_2_MAPb_2_I_7_ (0.2),^[^ ^32^ ^]^ (R‐/S‐PPA)EA_2_Pb_2_Br_7_ (0.3)^[^ ^33^ ^]^ et al. Such an APV‐driven large anisotropy factor shows excellent potential in high‐performance CPL detectors.

## 3 Conclusion

In summary, the circularly polarized light (CPL)‐dependent anomalous photovoltaic effect (APVE) is first realized in chiral hybrid perovskite, critical for photoelectric and photovoltaic fields. Precisely, by introducing lone‐pair electrons into organic layers, novel ACI chiral‐polar perovskites [R‐PPA][MOPA]PbBr_4_ (**2‐R**) (PPA = phenylpropylammonium MOPA = 3‐methoxypropylammonium) with large polarity are constructed, which exhibit anomalous photovoltaic of 6.50 V. Importantly, based on its intrinsic chiroptical activity, **2‐R** exhibits a unique CPL‐dependent APVE, which serves as a significant driving force, enabling exceptional self‐powered UV CPL detection with a high anisotropy factor (*g*_Iph_) of 0.33. This pioneering work provides new insights into designing anomalous photovoltaic detectors for future practical applications.

## Conflict of Interest

The authors declare no conflict of interest.

## Supporting information



Supporting Information

## Data Availability

The data that support the findings of this study are available from the corresponding author upon reasonable request.
