# Supplementary material for: Unprecedented Ultraviolet Circularly Polarized Light‐Dependent Anomalous Photovoltaics in Chiral Hybrid Perovskites
Source: Adv Sci (Weinh). 2025 Jan 15;12(10):2412506. doi: 10.1002/advs.202412506 (PMC11904972; doi:10.1002/advs.202412506)
Supplement: Supplementary file 1 — Supporting Information [file ADVS-12-2412506-s001.docx]

Supporting Information

Unprecedented Ultraviolet Circularly Polarized Light-Dependent Anomalous Photovoltaics in Chiral Hybrid Perovskites

*Qianwen Guan, Peng Xu, Bohui Xu, Huang Ye, Zeng-Kui Zhu, Shiyu Wang, Chengshu Zhang, Hang Li, Chengmin Ji, Zheshuai Lin, and Junhua Luo**

**Experimental Section**

**Materials**. (*S*)-(-)-1-Phenylpropylamine (C_9_H_13_N, 98%, Aladdin), (*R*)-(+)-1-Phenylpropylamine (C_9_H_13_N, 98%, Aladdin), lead(Ⅱ) acetate trihydrate Pb(Ac)_2_·3H_2_O (99.5%, Aladdin), hydrobromic acid (HBr, 48%, SCR), Propylamine (C_3_H_9_N, 98%, Aladdin), and 3-Methoxypropylamine (C_4_H_11_NO, 99%). All the chemicals were bought and used without any further purification.

**Synthesis and crystal growth:**

The synthesis of 2D (R/S-PPA)(MOPA)PbBr_4_ (**2-R/S**): Single crystals of **2-R** (or **2-S**) were obtained from a mixture of saturated HBr (47%, 20 mL) solution containing Pb(CH_3_COO)_2_·3H_2_O (2.00 g), *R*-(+)-phenypropylamine (or *S*-phenypropylamine) (0.27 g), and 3-Methoxypropylamine (99%, 2 mL). Then, the mixture was stirred for 30 minutes at room temperature to obtain a clear solution. Bulk crystals of **2-R** and **2-S** were grown by slowly cooling the above solution from 30 ℃ to 10 ℃ at a cooling rate of 1 ℃ per day.

The synthesis of 2D (R-PPA)PAPbBr_4_ (**1-R/S**): Single crystals of **1-R** (or **1-S**) were obtained from a mixture of saturated HBr (47%, 20 mL) solution containing Pb(CH_3_COO)_2_·3H_2_O (2.00 g), *R*-(+)-phenypropylamine (or *S*-phenypropylamine) (0.27 g), and propylamine (98%, 2 mL). Then, the mixture was stirred for 30 minutes at room temperature to obtain a clear solution. Bulk crystals of **1R** and **1S** were grown by slowly cooling the above solution from 30 ℃ to 10 ℃ at a cooling rate of 1 ℃ per day.

**Powder X-ray diffraction:** Powder X-ray diffraction (PXRD) of **1-R/S** and **2-R/S** was performed on a Rigaku MiniFlex diffractometer with a Cu Kα radiation source at room temperature. The diffraction patterns were collected in the 2*θ* range of 5° to 45° with a step size of 0.02°. The experimental PXRD patterns match well with the simulated data calculated by the single-crystal structures, which confirms the high purity of **1-R/S** and **2-R/S**.

**Single crystal X-ray Diffraction.** Single-crystal X-ray diffraction data were collected on a Bruker D8 diffractometer at room temperature using monochromatic Mo Kα radiation (λ = 0.77 Å). The unit cell measurement, data collection, integration, scaling, and absorption corrections for these data were done using Bruker Apex III software. The crystal structures of **2-R** and **2-S** were solved by direct methods and then refined by the full-matrix least-squares method using the SHELXLTL 2018 software package. The detailed crystallographic data and the structure refinement parameters of **2-R** and **2-S** have been listed in **Table S1**.

**Second harmonic generation (SHG) measurements.** The SHG signals were obtained on the pellets compressed from powder samples of **1-R** and **2-R**, using KH_2_PO_4_ (KDP) as a reference material (pulsed Nd:YAG at a wavelength of 1064 nm, 5 ns pulse duration, 1.6 MW peak power, 10 Hz repetition rate).

**Optical measurements.** A Lambda950 Ultraviolet-Visible (UV-Vis) spectrophotometer (PerkinElmer) was used for UV-Vis diffuse reflection measurements with BaSO_4_ as the 100% reflectance reference.

**Circular dichroism (CD).** Chiral compounds were mixed with KBr in a ratio of 1 to 50 and fully ground to get powder samples. For CD tests, 34 mg of each mixed powder sample is pressed into uniform, transparent pellets. A Bio-Logic MOS450 CD spectrometer was used for the solid-state CD measurements. The background was pure KBr pellets, and the spectra were obtained at a scan rate of 200 nm/min, with the data pitch being 1 nm.

**Computational description.** CASTEP performed the first-principles calculations of **2-R** based on density functional theory (DFT). PBESOL treated the exchange-correlation effects in the generalized gradient approximation. The norm-conserving pseudopotentials in the Kleinman-Bylander form were adopted to model the interactions between ionic cores and electrons.

**Photoelectric measurements.** The planer-type photodetectors were built on single crystals' (00l) surface. The current signals were collected using a Keithel 6517B electrometer. A laser diode with a wavelength of 377 nm was used as a light source. Circularly polarized light was obtained by a linear polarizer (Thorlabs) and a quarter-wave plate (Thorlabs). A standard Si detector calibrated the intensity of light.

**Table S1** Crystal data of **2-R** and **2-S** collected at room temperature.

|  | **2-R** | **2-S** |
| --- | --- | --- |
| Empirical formula | C_13_H_26_Br_4_N_2_OPb | C_13_H_26_Br_4_N_2_OPb |
| Formula weight | 753.19 | 753.19 |
| Temperature (K) | 298.39 | 299.74 |
| Crystal system | monoclinic | monoclinic |
| Space group | *P*2_1_ | *P*2_1_ |
| *a* (Å) | 8.0720(5) | 8.0586(10) |
| *b* (Å) | 8.4098(6) | 8.3991(9) |
| *c* (Å) | 16.9527(12) | 16.939(2) |
| *α* (°) | 90 | 90 |
| *β* (°) | 102.897(3) | 102.974(5) |
| *γ* (°) | 90 | 90 |
| *V* (Å^3^) | 1121.78(13) | 1117.2(2) |
| *Z* | 2 | 2 |
| *ρ*_calc_ (g/cm^3^) | 2.230 | 2.239 |
| *F*(000) | 696.0 | 696.0 |
| Radiation *λ* (Mo Kα) (Å) | 0.71073 | 0.71073 |
| 2*θ* range for data collection (°) | 4.93 to 55.132 | 5.188 to 55.08 |
| Index ranges | -10 ≤ *h* ≤ 10,  -10 ≤ *k* ≤ 10,  -22 ≤ *l* ≤ 22 | -10 ≤ *h* ≤ 10,  -10 ≤ *k* ≤ 10,  -21 ≤ *l* ≤ 21 |
| Reflections collected | 22398 | 20005 |
| Independent reflections | 5154 [*R*_int_ = 0.0650,  *R*_sigma_ = 0.0587] | 5127 [*R*_int_ = 0.1014,  *R*_sigma_ = 0.0855] |
| Data/restraints/parameters | 5154/120/182 | 5127/103/194 |
| Goodness-of-fit on *F*^2^ | 1.063 | 1.018 |
| Final R indexes [*I* >= 2*σ* (*I*)] | *R*_1_ = 0.0409,  *wR*_2_ = 0.1152 | *R*_1_ = 0.0494,  *wR*_2_ = 0.0914 |
| Final R indexes [all data] | *R*_1_ = 0.0532,  *wR*_2_ = 0.1246 | *R*_1_ = 0.0904,  *wR*_2_ = 0.1094 |
| Flack parameter | 0.037(10) | 0.017(17) |

^a^R_1_ = Σ||F_o_| – |F_c_||/Σ|F_o_|, wR_2_ = [Σw(F_o_^2^ – F_c_^2^)^2^/Σ(F_o_^2^)^2^]^1/2^

**
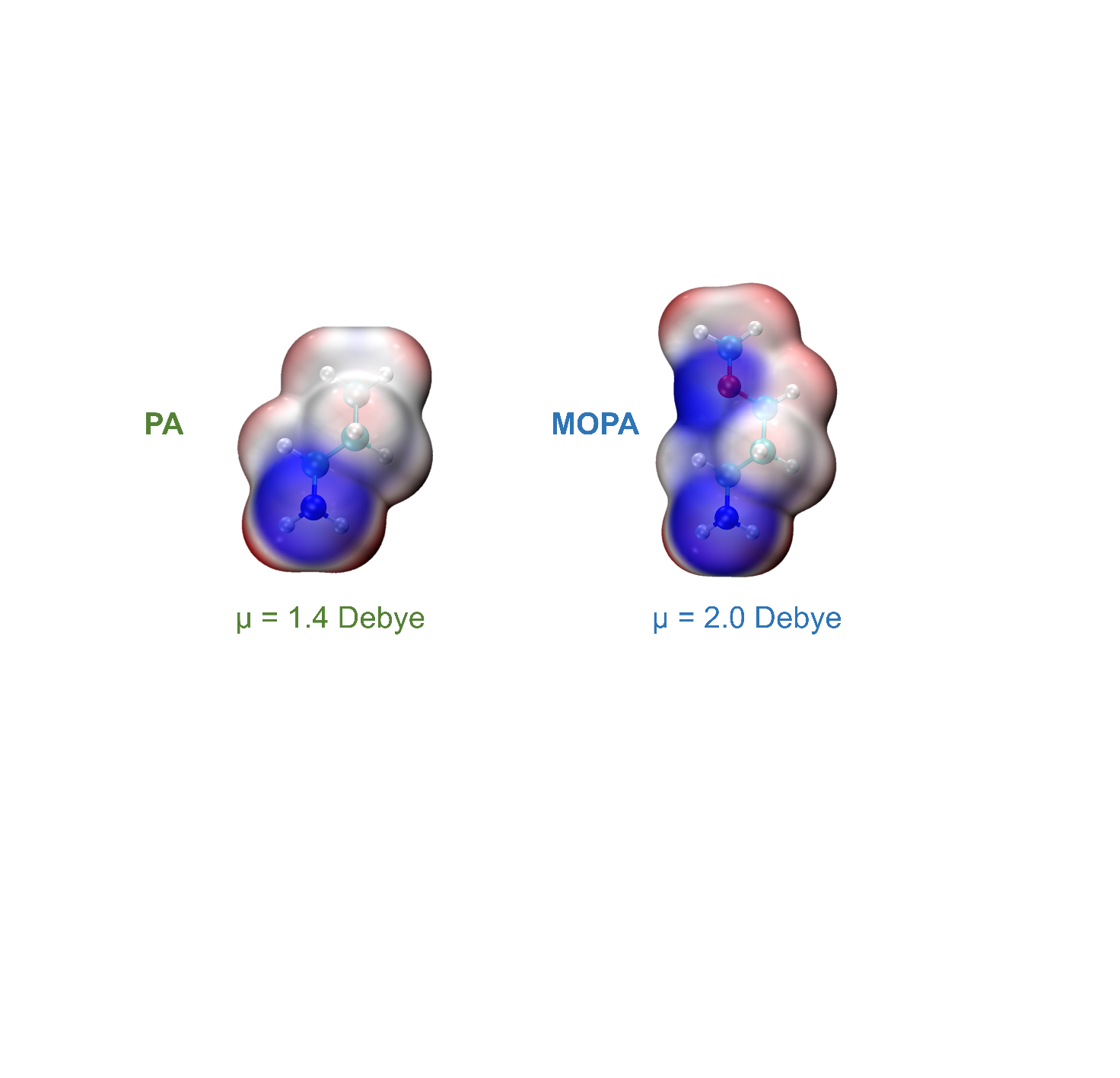
**

**Figure S1.** The dipole moment of PA and MOPA molecules.

**
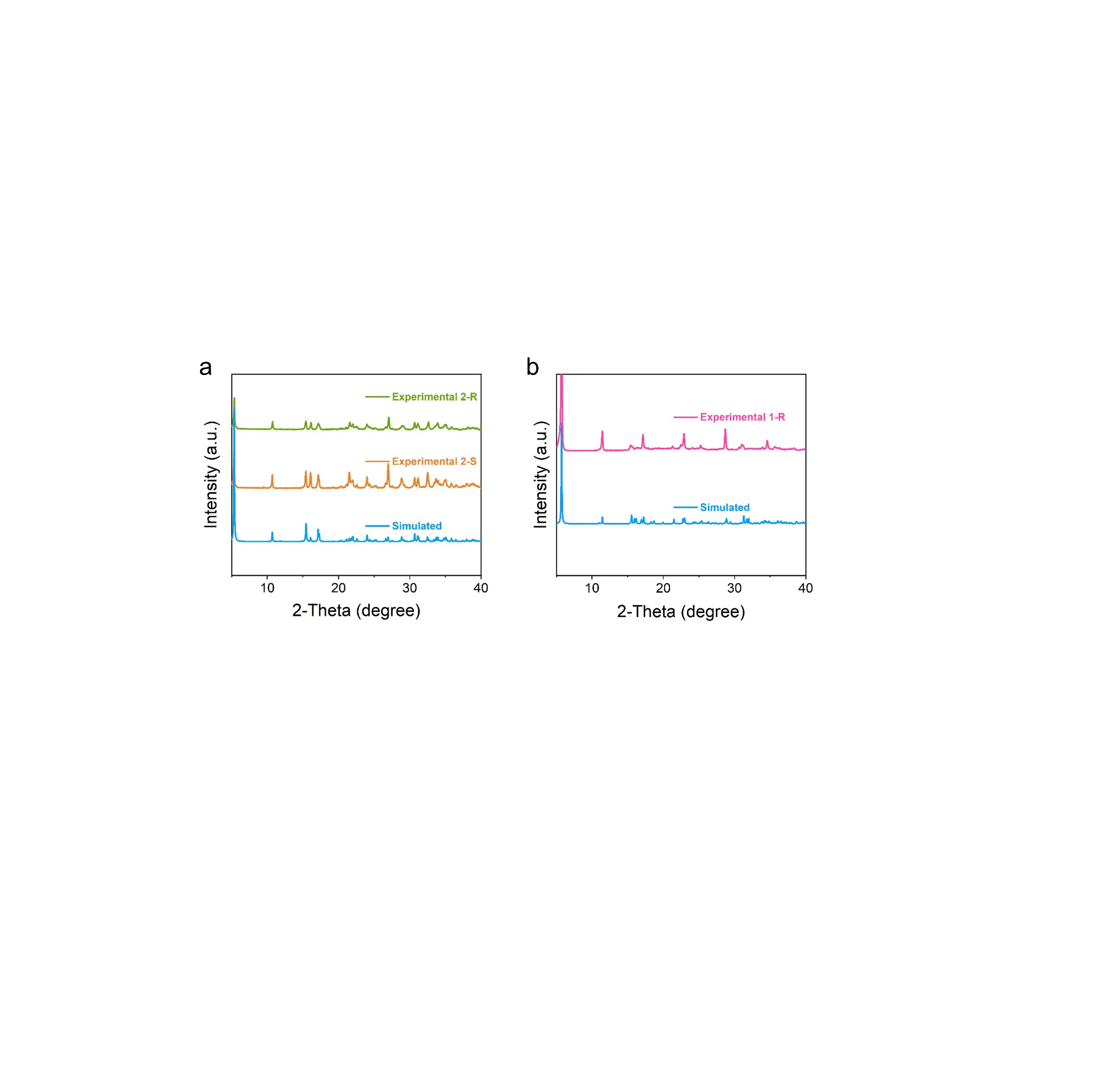
**

**Figure S2.** Experimental and calculated powder X-ray diffraction patterns of (a)**2-R/S** and (b)**1-R**.

**
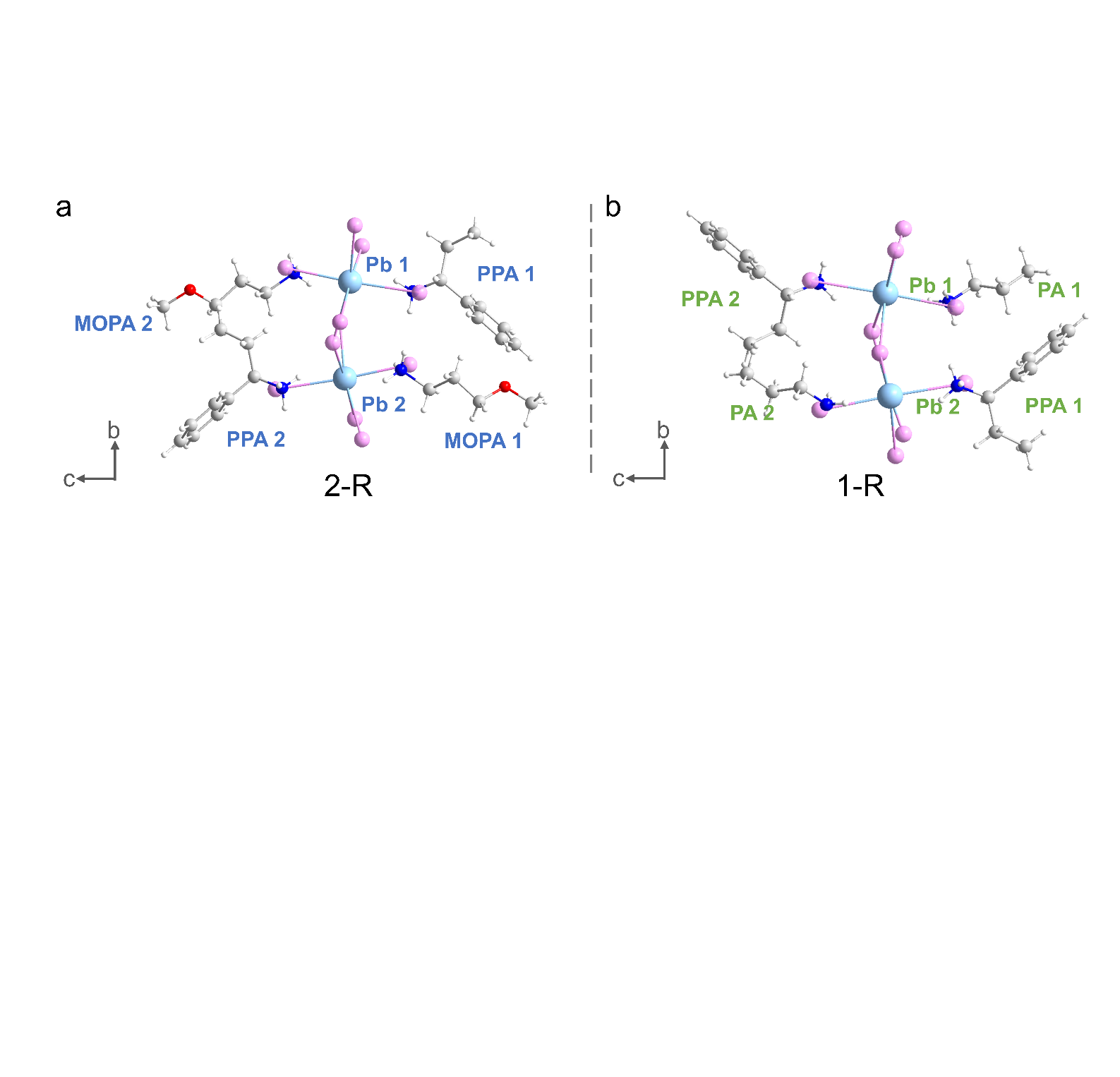
**

**Figure S3.** (a) The minimum repetition unit of **2-R** and (b) **1-R** in crystals.

**Table S2.** The dipole moment calculation of the inorganic skeleton and cations (Figure S3).

| Dipole moment (Debye) | | | | | |
| --- | --- | --- | --- | --- | --- |
| Crystal cell | Species | X | Y | Z | dipole moment |
| (R-PPA)PAPbBr_4_ | PA1 | -1.1574 | -2.0173 | -5.3047 | 5.7921 |
|  | PA2 | 1.1574 | -2.0173 | 5.3047 | 5.7921 |
|  | PPA1 | -0.6492 | 1.3530 | 7.1794 | 7.3346 |
|  | PPA2 | 0.6492 | 1.3530 | -7.1794 | 7.3346 |
|  | Total Organic | 0.0000 | -1.3284 | 0.0000 | **1.3284** |
|  | [Pb_1_Br_6_] | 2.6651 | 2.5534 | 12.0847 | 12.6358 |
|  | [Pb_2_Br_6_] | -2.6651 | 2.5534 | -12.0847 | 12.6358 |
|  | Total Inorganic | 0.0000 | 5.1069 | 0.0000 | **5.1069** |
|  | Total Unit | 0.0000 | 3.7784 | 0.0000 | 3.7784 |
|  | Total crystal | 0.0000 | 7.5568 | 0.0000 | **7.5568** |
| (R-PPA)  (MOPA)PbBr_4_ | MOPA1 | 4.0086 | 2.1058 | 9.4686 | 10.4956 |
|  | MOPA2 | -4.0086 | 2.1058 | -9.4686 | 10.4956 |
|  | PPA1 | -0.0485 | 1.4368 | 7.4508 | 7.5882 |
|  | PPA2 | 0.0485 | 1.4368 | -7.4508 | 7.5882 |
|  | Total Organic | 0.0000 | 7.0853 | 0.0000 | **7.0853** |
|  | [Pb_1_Br_6_] | -11.5925 | 8.2496 | 3.0269 | 14.5466 |
|  | [Pb_2_Br_6_] | 11.5939 | 8.2484 | -3.0269 | 14.570 |
|  | Total Inorganic | 0.0014 | 16.4980 | 0.0003 | **16.4980** |
|  | Total Unit | 0.0014 | 23.5833 | 0.0003 | 23.5833 |
|  | Total crystal | 0.0014 | 23.5833 | 00003 | **23.5833** |

**
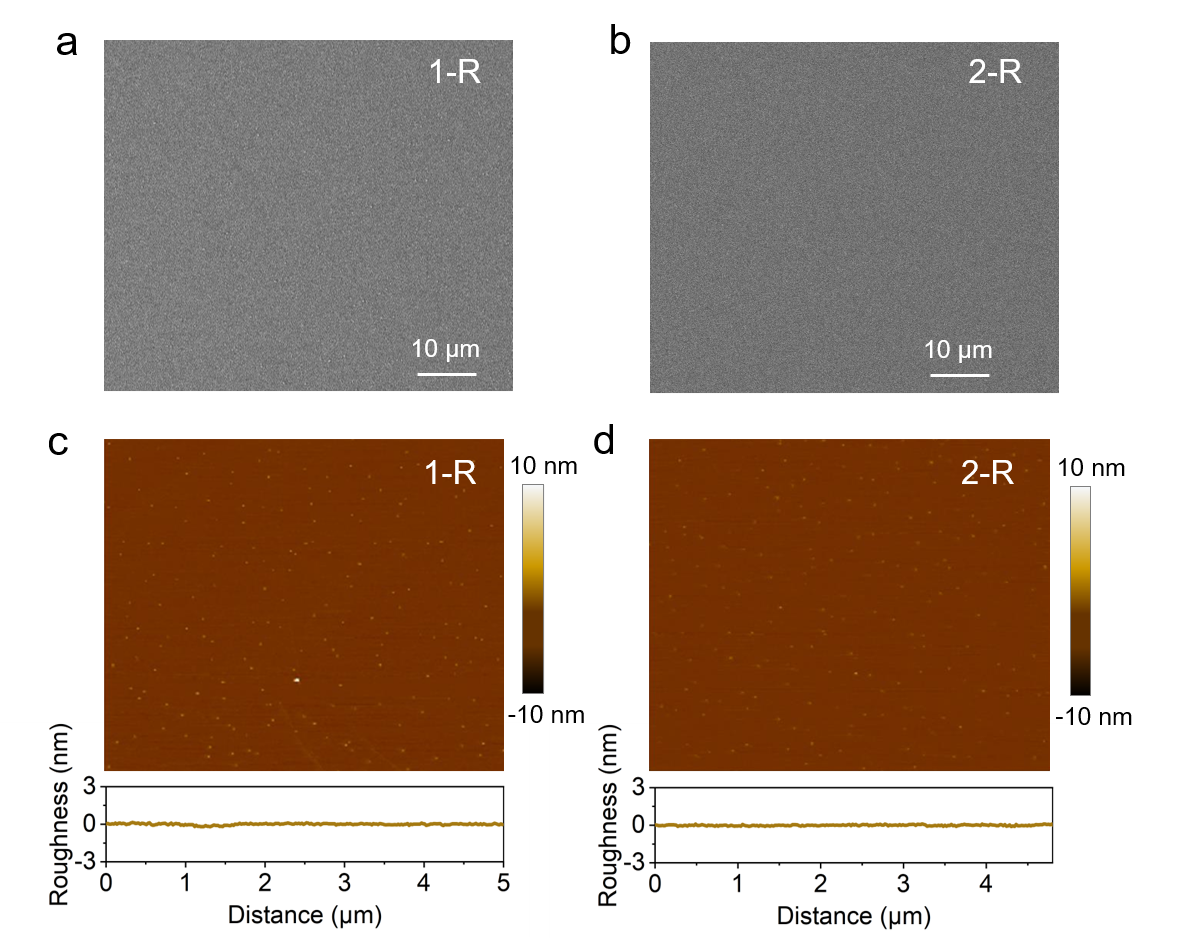
**

**
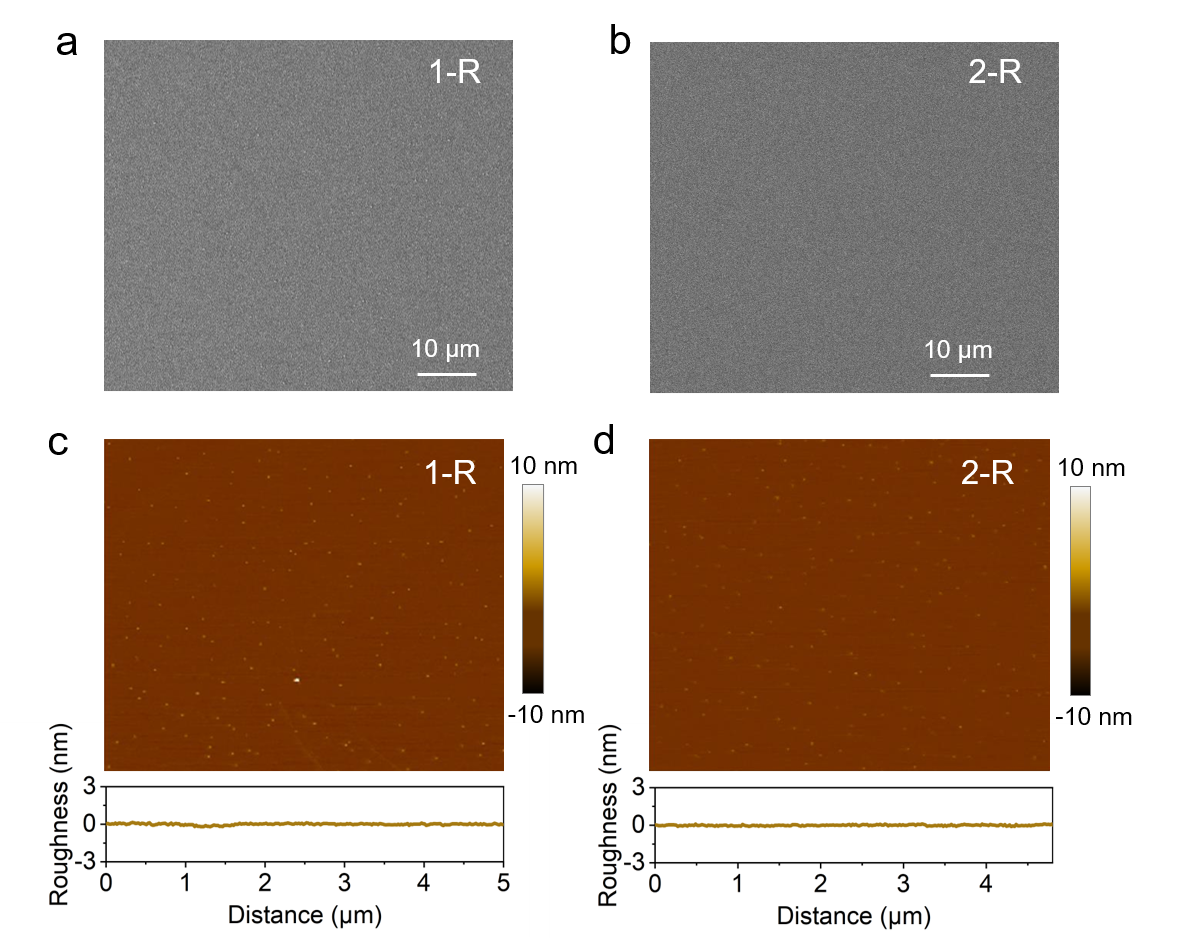
**

**Figure S4.** (a) The SEM spectra of **1-R** and (b) **2-R**. (c). The AFM spectra of **1-R** (Ra is 0.0679 nm and Rq is 0.134 nm) and (d) **2-R** (Ra is 0.0833 nm and Rq is 0.212 nm).

**
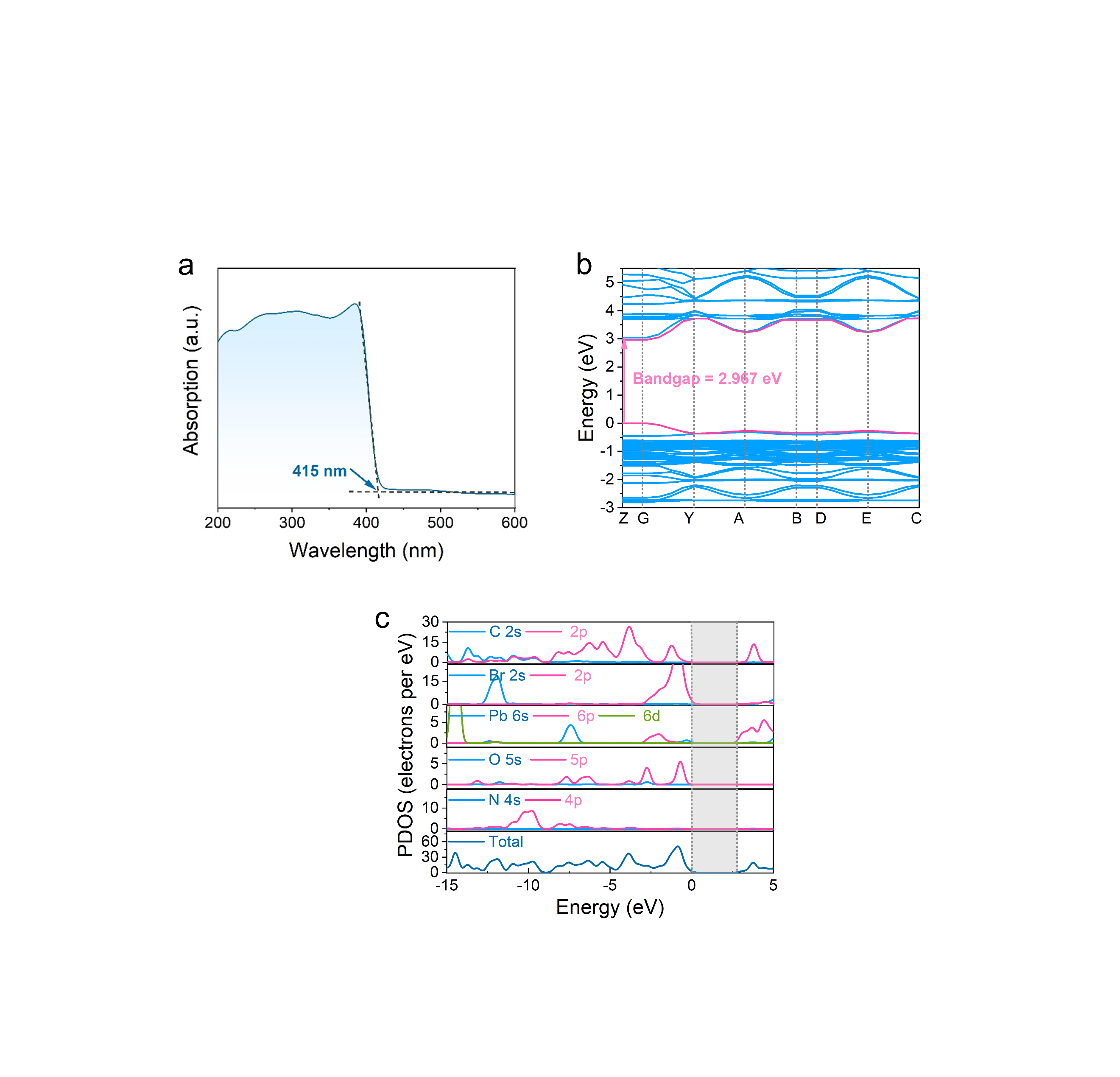
**

**
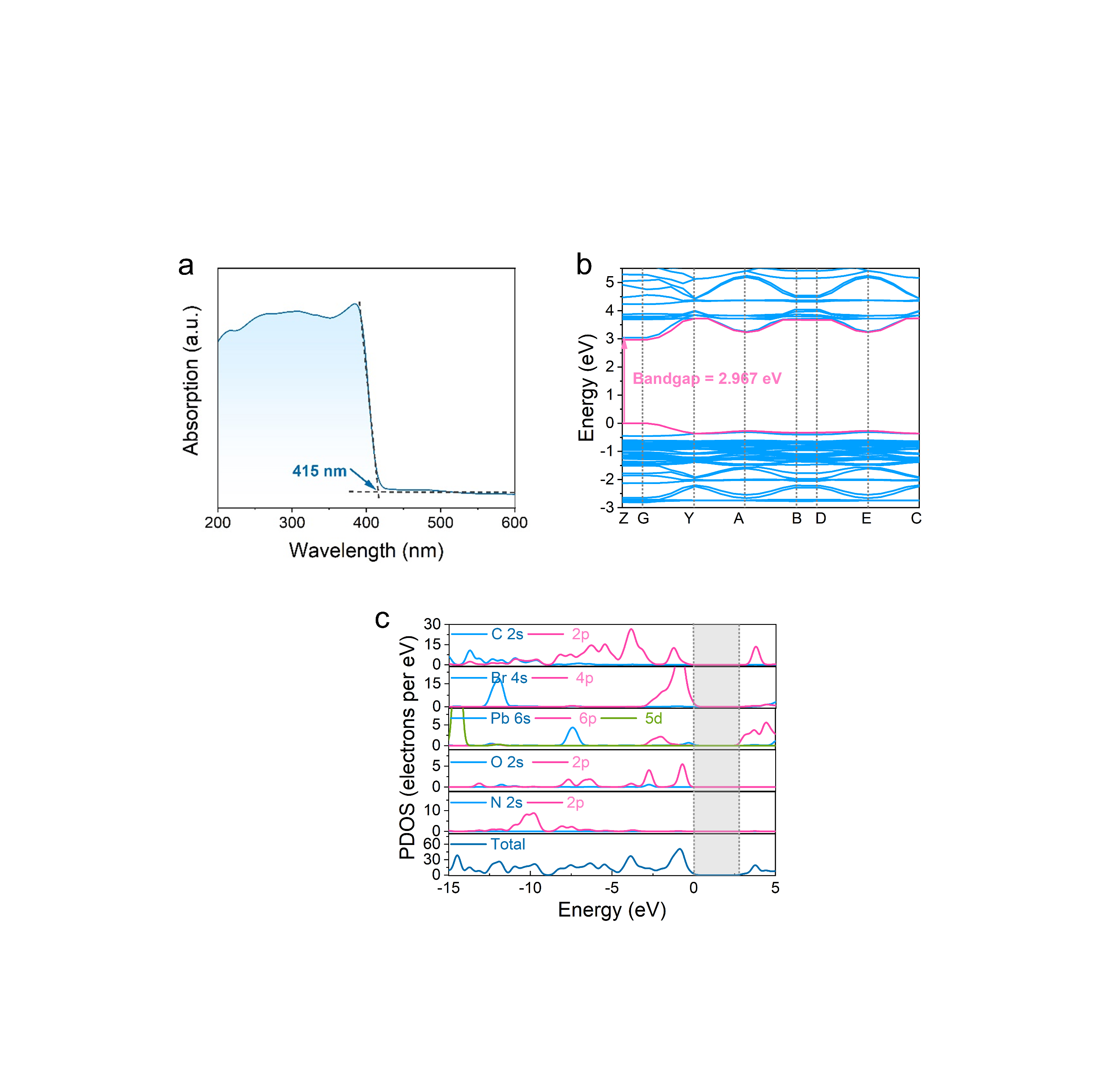
**

**Figure S5.** (a) The absorption edge of **2-R**. (b). The DFT calculation of **2-R**.(c) The partial density of states of **2-R**.

**
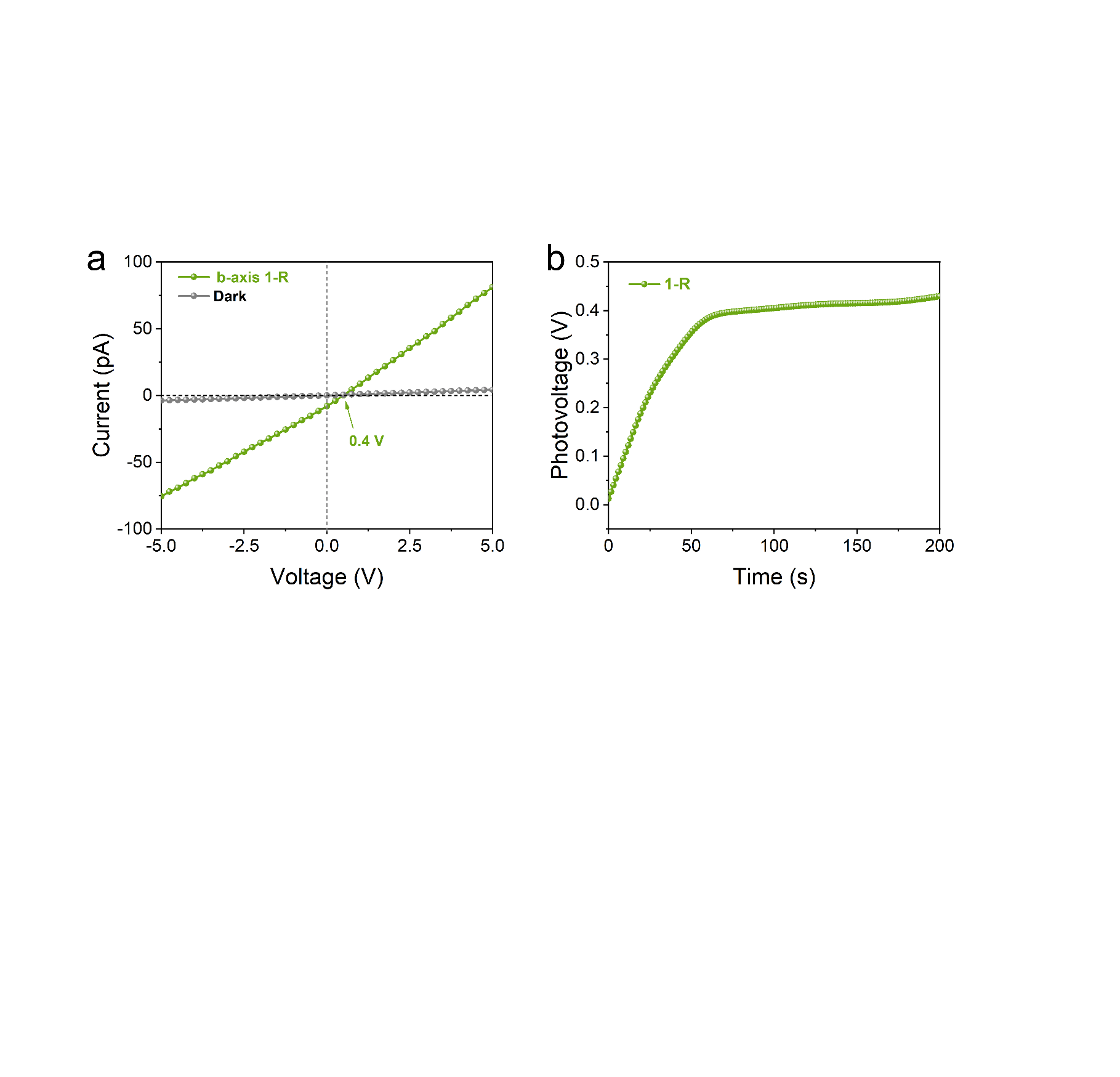
**

**Figure S6.** The photovoltage of **1-R**.

**Table S3.** The photovoltages of the reported OIHPs and this work.

| **Material** | **Bandgap/eV** | **Photovoltage/V** | **Ref.** |
| --- | --- | --- | --- |
| (*R*-PPA)(EA)PbCl_4_ | 3.5 | 0.5 | ^[1]^ |
| (*R*-PPA)(EA)PbBr_4_ | 3.05 | 2.5 | ^[2]^ |
| (SMBA)_2_Pb_0.9_Sn_0.1_I_4_ | 2.36 | 0.4 | ^[3]^ |
| (*R*-MPA)_4_AgBiI_8_ | 2.01 | 0.068 | ^[4]^ |
| (R/S-3AMP)PbBr_4_ | 2.78 | 2.5 | ^[5]^ |
| (S-β-MPA)EAPbBr_4_ | 3.16 | 0.36 | ^[6]^ |
| (R-PPA)(PA)PbBr_4_ | 3.01 | 0.23 | ^[7]^ |
| [(R)-b-MPA]_4_AgBiI_8_ | 2.01 | 0.068 | ^[4]^ |
| S/R-[(4AMPEA]_2_AgBiI_8_·0.5H_2_O | 1.96 | 0.080 | ^[8]^ |
| (S-BPEA)_2_FAPb_2_I_7_ | 1.6 | 0.85 | ^[9]^ |
| (R-MPz)_6_Bi_3_I_21_･6H_2_O | 1.94 | 1.1 | ^[10]^ |
| (R,S-MBA_0.5_nBA_0.5_)_2_PbI_4_ | 2.4 | 0.63 | ^[11]^ |
| **2-R** | **3.01** | **6.50** | **This work** |


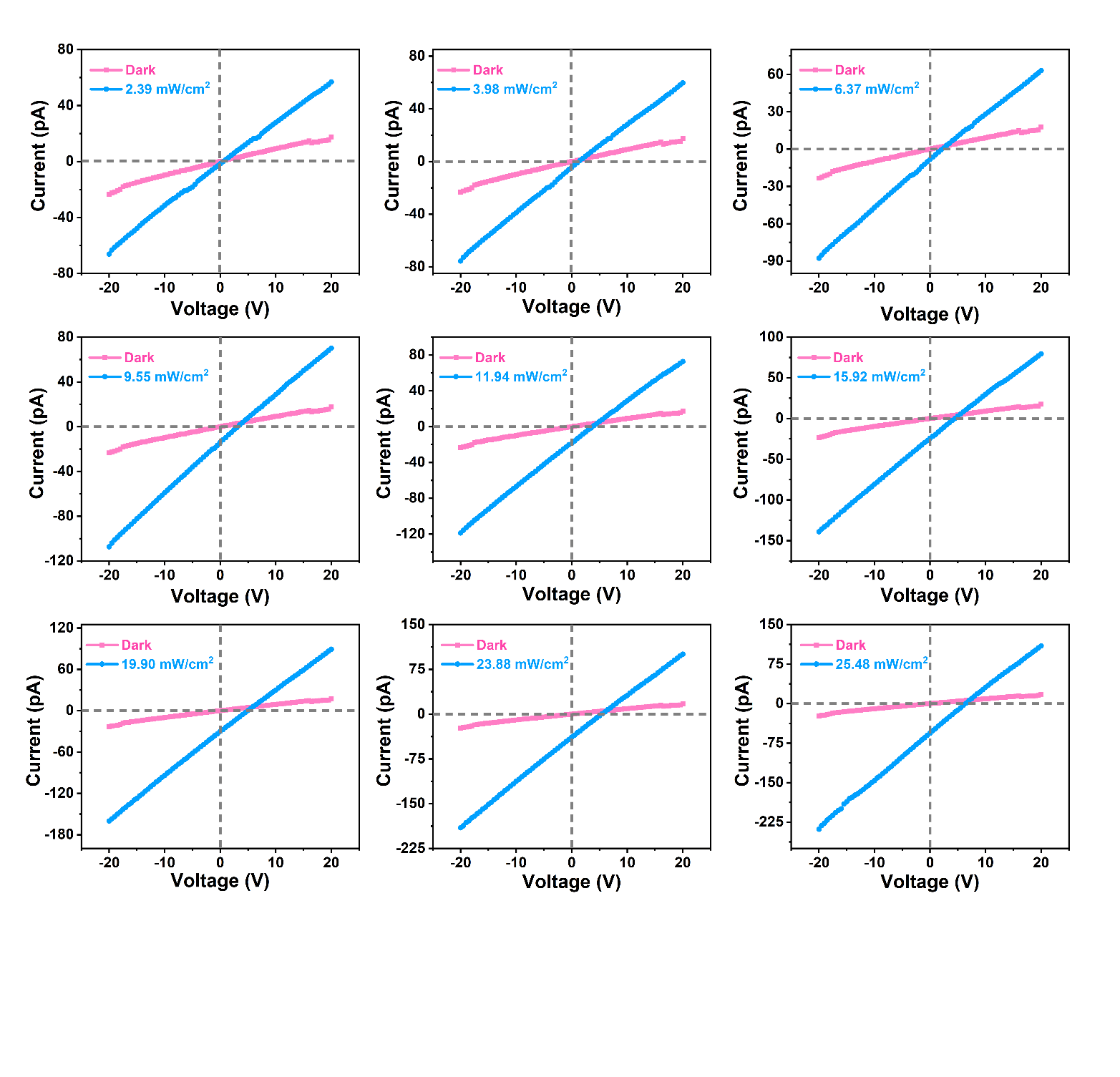


**Figure S7.** The I-V curves of **2-R** under different intensities of 377 nm light illumination.

**
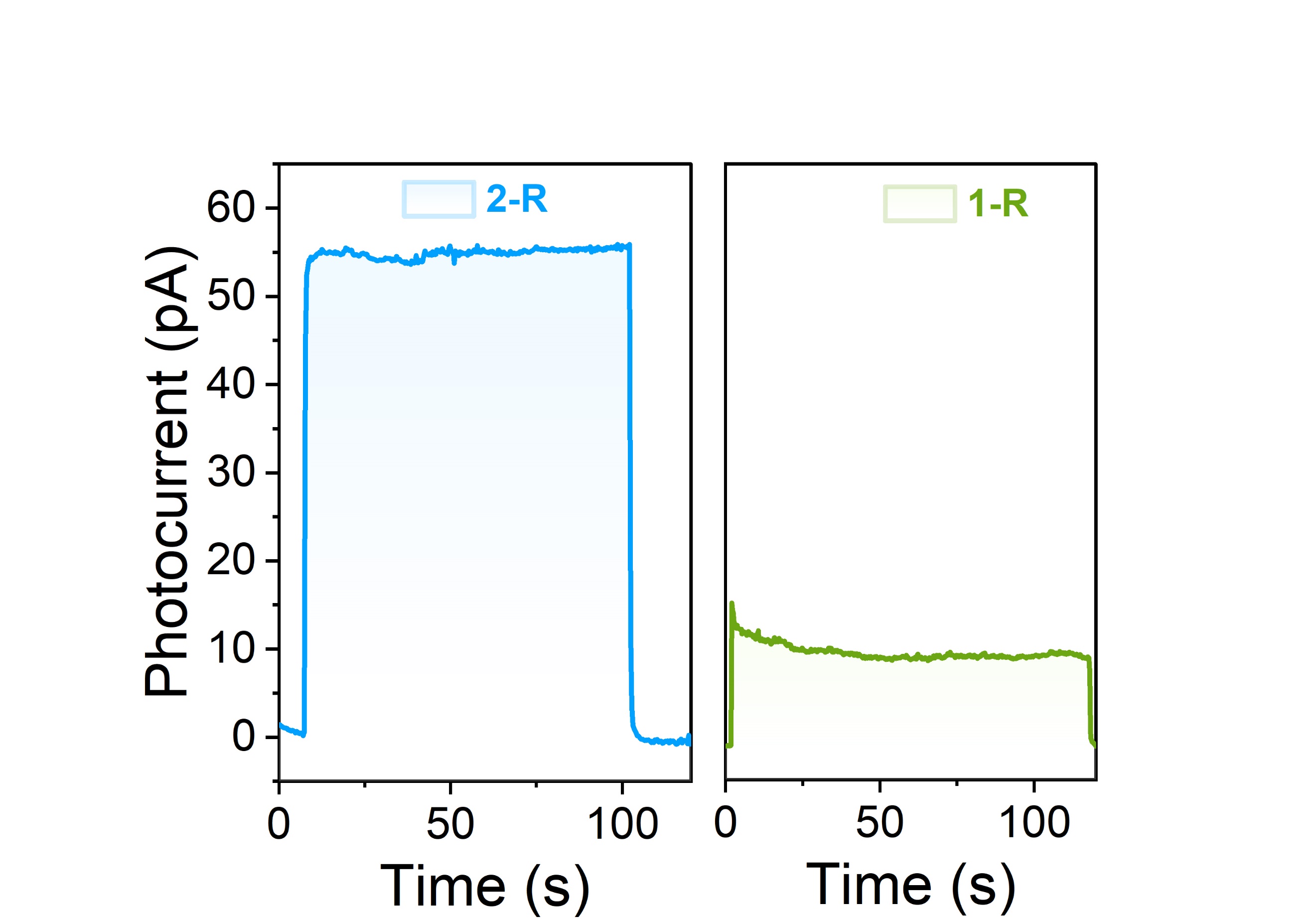
**

**Figure S8.** The photo responses of **2-R** and **1-R** under 25.5 mW/cm^2^.

**
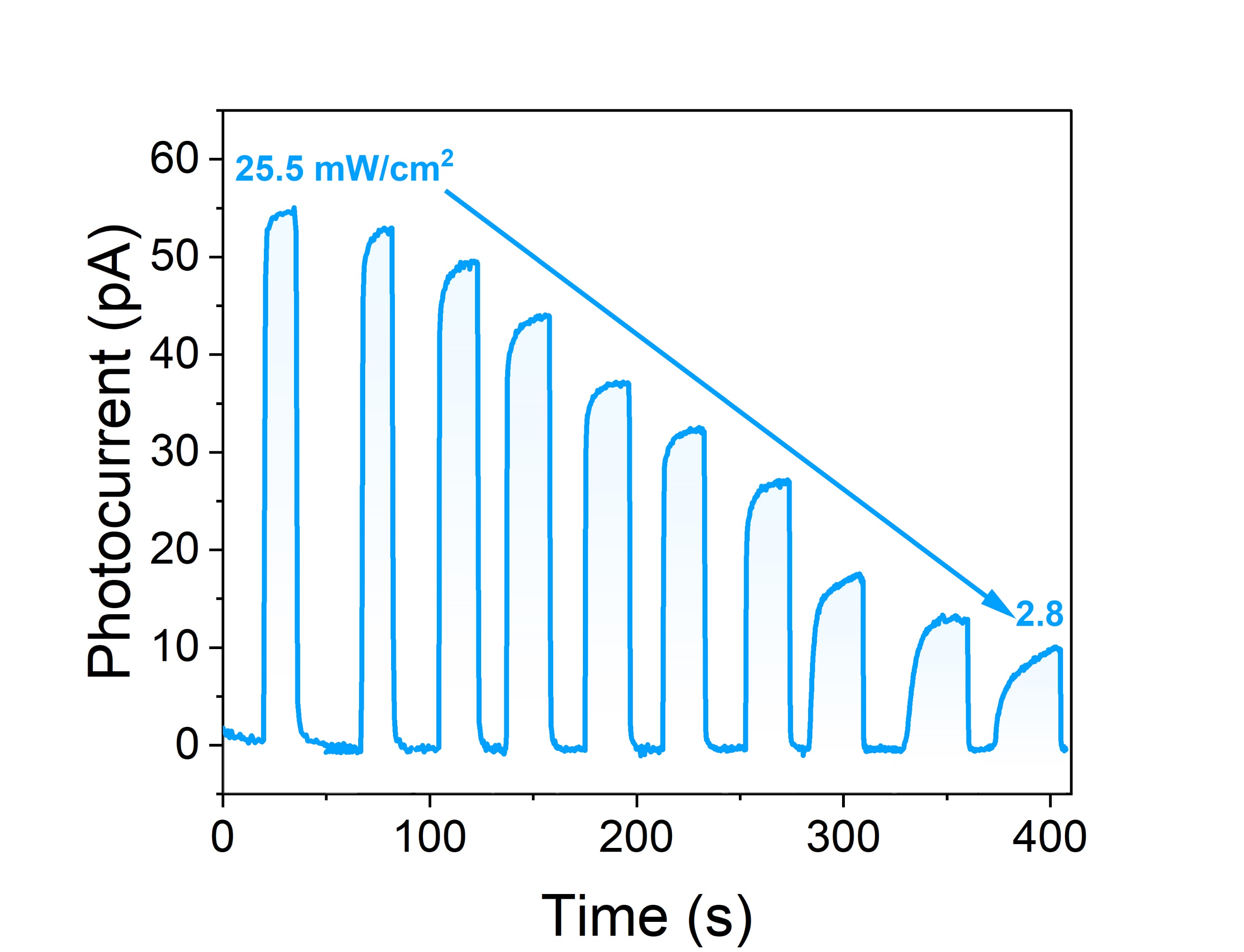

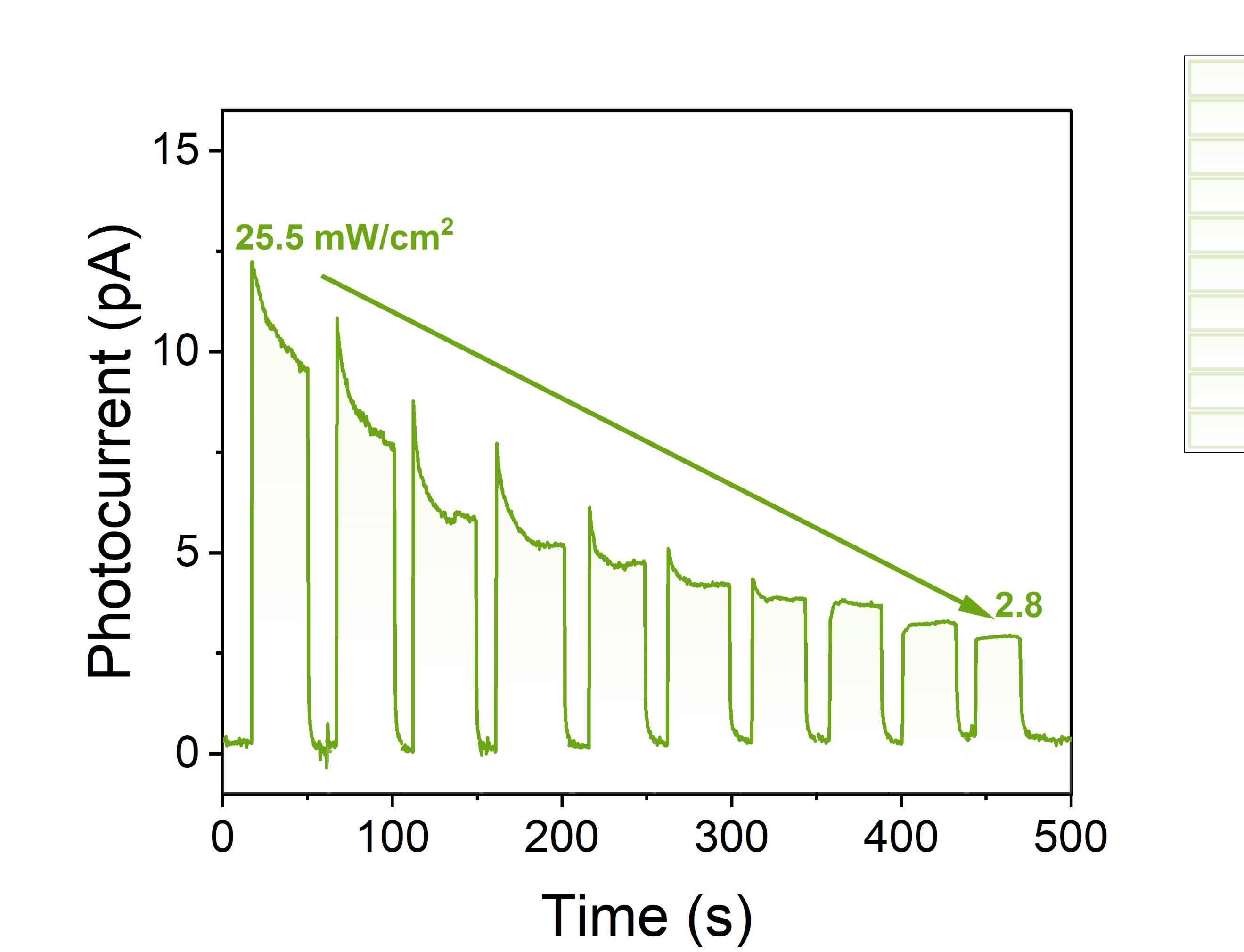

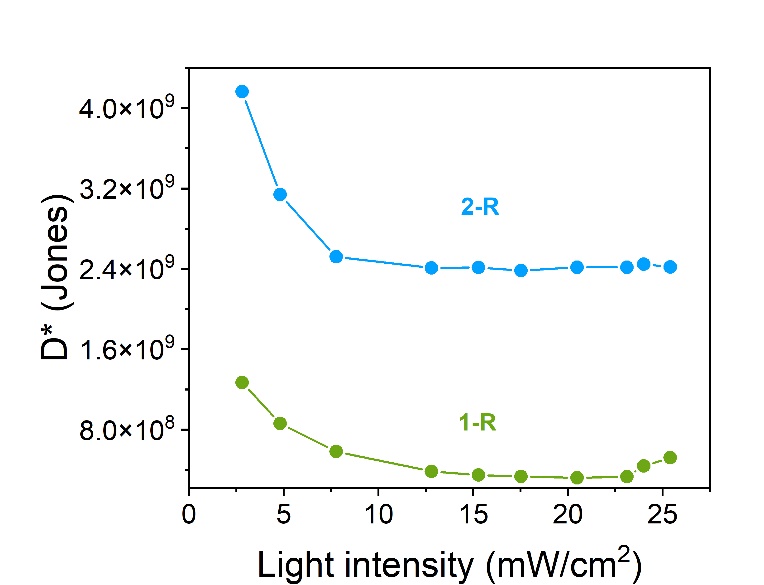

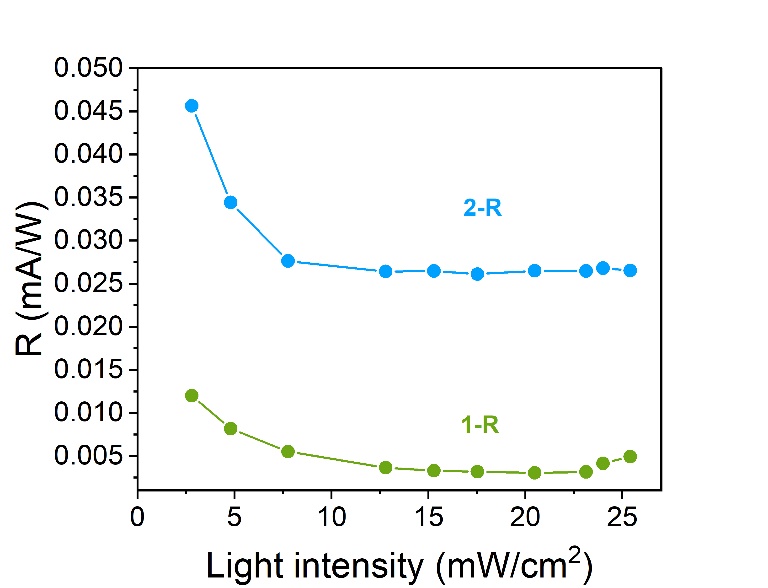
**

**Figure S9.** The photo responses of **2-R** and **1-R**.

**
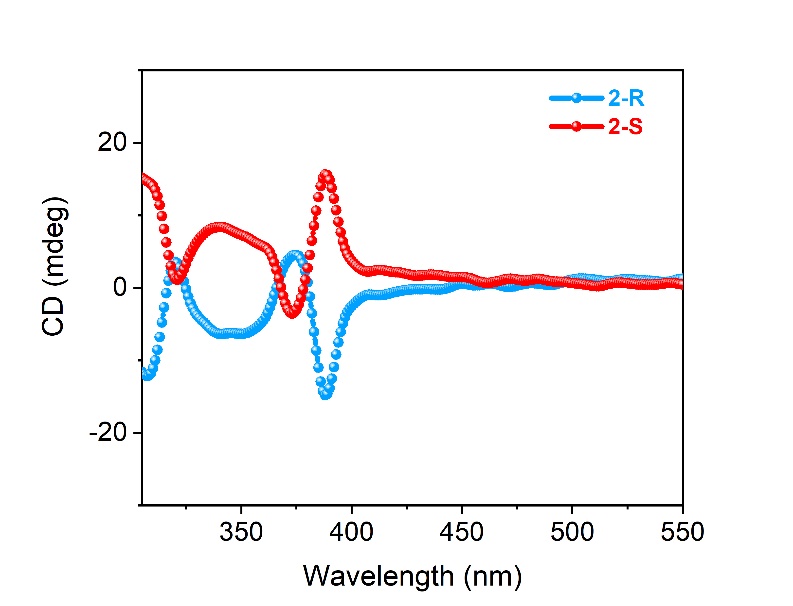
**

**Figure S10.** The circular dichroism spectra of **2-R/S**

**Table S4.** The chiral perovskites CPL detectors in this work are compared to the reported CPL detectors.

| **Materials** | **Dimension** | **Anisotropy factor**  **(g_Iph_)@wavelength** | **Power** | **Ref.** |
| --- | --- | --- | --- | --- |
| [(*R*)-β-MPA]_4_AgBiI_8_ single crystal (SC) | 2D | 0.3@520nm | 0 V | ^[12]^ |
| (R-/S-PPA)EA_2_Pb_2_Br_7_ SC | 2D | 0.3@266nm | 0 V | ^[13]^ |
| (SPPA)_4_(IPA)_6_Ag_2_Bi_4_I_24_⋅2H_2_O SC | 2D | 0.21@520nm | 1 V | ^[14]^ |
| (*R*-PPA)EAPbCl_4_ SC | 2D | 0.4@266nm | 0 V | ^[1]^ |
| (R-PPA)EAPbBr_4_ SC | 2D | 0.42@266nm | 0 V | ^[2]^ |
| (*R*-*β*-MPA)EAPbBr_4_ SC | 2D | 0.19@405nm | 0 V | ^[6]^ |
| (*R*-BPEA)_2_PbI_4_ SC | 2D | 0.13@520nm | 10 V | ^[15]^ |
| (*R*/*S*-3AMP)PbBr_4_ SC | 2D | 0.20@430 nm | 10 V | ^[5]^ |
| (*R*/*S*-BPEA)EA_6_Pb_4_Cl_15_ SC | 3D | 0.28@320nm | 10 V | ^[16]^ |
| (4-AMP)BiI_5_ SC | 1D | 0.24@405nm | 0 V | ^[17]^ |
| (*S*-α-MBA)_2_PbI_4_ NW array | 2D | 0.24@510nm | 5 V | ^[18]^ |
| [(*R*)-β-MPA]_2_MAPb_2_I_7_ film | 2D | 0.2@532nm | 5 V | ^[19]^ |
| (NEA)_2_(MA)_n‑1_Pb_n_I_3n+1_ film | 2D | 0.15@405nm | 20 V | ^[20]^ |
| (R-α-PEA)_2_PbI_4_ Nanowires | 2D | 0.15@505nm | 5 V | ^[21]^ |
| MAPbBr_3_-*R* | 3D | 0.39@405nm | 5 V | ^[22]^ |
| (R-NEA)PbI_3_ flake | 1D | 0.294@405nm | 4 V | ^[23]^ |
| (*R*-C_5_H_14_N)PbI_3_ microwire | 1D | 0.23@405nm | 5 V | ^[24]^ |

**References**

[1] T. Zhu, H. Wu, C. Ji, X. Zhang, Y. Peng, Y. Yao, H. Ye, W. Weng, W. Lin, J. Luo, *Adv. Opt. Mater.* **2022**, *10*, 2200146.

[2] T. Zhu, W. Weng, C. Ji, X. Zhang, H. Ye, Y. Yao, X. Li, J. Li, W. Lin, J. Luo, *J. Am. Chem. Soc.* **2022**, *144*, 18062 – 18068.

[3] B. Yao, Q. Wei, Y. Yang, W. Zhou, X. Jiang, H. Wang, M. Ma, D. Yu, Y. Yang, Z. Ning, *Nano Lett.* **2023**, *23*, 1938−1945.

[4] D. Li, X. Liu, W. Wu, Y. Peng, S. Zhao, L. Li, M. Hong, J. Luo, *Angew. Chem. Int. Ed.* **2021**, *60*, 8415-8418.

[5] C.-C. Fan, X.-B. Han, B.-D. Liang, C. Shi, L.-P. Miao, C.-Y. Chai, C.-D. Liu, Q. Ye, W. Zhang, *Adv. Mater.* **2022**, *34*, 2204119.

[6] W. Wu, L. Li, D. Li, Y. Yao, Z. Xu, X. Liu, M. Hong, J. Luo, *Adv. Opt. Mater.* **2022**, *10*, 2102678.

[7] Z.-K. Zhu, T. Zhu, S. You, P. Yu, J. Wu, Y. Zeng, Y. Jiang, X. Liu, L. Li, C. Ji, J. Luo, *Adv. Sci.* **2023**, 2307593.

[8] Z. Li, C. Ji, Y. Fan, T. Zhu, S. You, J. Wu, R. Li, Z.-K. Zhu, P. Yu, X. Kuang, J. Luo, *J. Am. Chem. Soc.* **2023**, *145*, 25134−25142.

[9] Q. Guan, H. Ye, S. You, Z.-K. Zhu, H. Li, X. Liu, J. Luo, *Small* **2023**, 2307908.

[10] X. Dong, T. Chen, J. Liang, L. Wang, H. Wu, Z. Xu, J. Luo, L.-N. Li, *Chin. J. Struct. Chem.* **2024**, *43*, 100256.

[11] X. Zhang, Y. Xu, A. N. Alphenaar, S. Ramakrishnan, Y. Zhang, A. J. Babatunde, Q. Yu, *ACS Nano* **2024**. *18*, 14605 - 14616.

[12] Y. Zhao, M. Dong, J. Feng, J. Zhao, Y. Guo, Y. Fu, H. Gao, J. Yang, L. Jiang, Y. Wu, *Adv. Opt. Mater.* **2022**, *10*, 2102227.

[13] T. Zhu, K. Zhang, C. Ji, X. Zhang, H. Ye, Y. Zou, J. Luo, *Small* **2022**, *18*, 2203571.

[14] Z.-K. Zhu, T. Zhu, J. Wu, S. You, P. Yu, X. Liu, L. Li, C. Ji, J. Luo, *Adv. Funct. Mater.* **2023**, *33*, 2214660.

[15] Y. Peng, X. Liu, L. Li, Y. Yao, H. Ye, X. Shang, X. Chen, J. Luo, *J. Am. Chem. Soc.* **2021**, *143*, 14077 – 14082.

[16] Q. Guan, T. Zhu, Z. K. Zhu, H. Ye, S. You, P. Xu, J. Wu, X. Niu, C. Zhang, X. Liu, J. Luo, *Angew. Chem. Int. Ed.* **2023**, *62*, e202307034.

[17] T. Zhu, J. Bie, C. Ji, X. Zhang, L. Li, X. Liu, X.-Y. Huang, W. Fa, S. Chen, J. Luo, *Nat. Commun.* **2022**, *13*, 7702.

[18] Z. Liu, C. Zhang, X. Liu, A. Ren, Z. Zhou, C. Qiao, Y. Guan, Y. Fan, F. Hu, Y. S. Zhao, *Adv. Sci.* **2021**, *8*, 2102065.

[19] L. Wang, Y. Xue, M. Cui, Y. Huang, H. Xu, C. Qin, J. Yang, H. Dai, M. Yuan, *Angew. Chem. Int. Ed* **2020**, *59*, 6442-6450.

[20] T. Liu, W. Shi, W. Tang, Z. Liu, B. C. Schroeder, O. Fenwick, M. J. Fuchter, *ACS Nano* **2022**, *16*, 2682−2689.

[21] Y. Zhao, Y. Qiu, J. Feng, J. Zhao, G. Chen, H. Gao, Y. Zhao, L. Jiang, Y. Wu, *J. Am. Chem. Soc.* **2021**, *143*, 8437−8445.

[22] G. Chen, X. Liu, J. An, S. Wang, X. Zhao, Z. Gu, C. Yuan, X. Xu, J. Bao, H.-S. Hu, J. Li, X. Wang, *Nat. Chem.* **2023**, *15*, 1581–1590.

[23] M. Li, F. Fang, X. Huang, G. Liu, Z. Lai, Z. Chen, J. Hong, Y. Chen, R.-j. Wei, G.-H. Ning, K. Leng, Y. Shi, B. Tian, *Chem. Mater.* **2022**, *34*, 2955−2962.

[24] Y. Zhao, X. Li, J. Feng, J. Zhao, Y. Guo, M. Yuan, G. Chen, H. Gao, L. Jiang, Y. Wu, *Giant* **2021**, *9*, 100086.
